# La fibrose rétropéritonéale idiopathique: une cause rare de douleurs lombaires chez le sujet âgé

**DOI:** 10.11604/pamj.2015.21.143.7239

**Published:** 2015-06-23

**Authors:** Olfa Berriche, Maher Dhifallah

**Affiliations:** 1 Service de Médecine Interne, Hôpital Mahdia, Mahdia, Tunisie; 2 Service de Radiologie, Hôpital Mahdia, Mahdia, Tunisie

**Keywords:** Fibrose, âgé, douleurs, Fibrosis, elderly, pain

## Image en medicine

La fibrose rétropéritonéale (FRP) est une maladie rare, caractérisée par une transformation du tissu rétropéritonéal en une nappe sclérofibreuse rétractile, d'origine inflammatoire associée à un engainement des uretères et des organes adjacents dont l’étiopathogénie reste mal comprise. Elle est dotée d'un grand polymorphisme clinique, d'une disparité étiologique considérable et elle est caractérisée par l'absence de consensus thérapeutique. Nous rapportons l'observation d'une patiente âgé de 63 ans, se plaignant de coliques néphrétiques rebelles au traitement antalgique trainantes et inexpliquées pendant 3 mois, elle a bénéficié d'un uro scanner ayant montré un aspect en faveur d'une FRP compliquée d'une sténose urétérale droite et d'une obstruction urétérale complète à gauche, elle a bénéficié d'une montée de sonde JJ. Le bilan étiologique (traumatisme, prise médicamenteuse, connectivite, cause infectieuse, cause néoplasique…) était négatif. La patiente a bénéficié d'une corticothérapie orale à fortes doses, l’évolution ultérieure était marquée par l'amélioration partielle de la symptomatologie clinique et des signes radiologiques.

**Figure 1:**
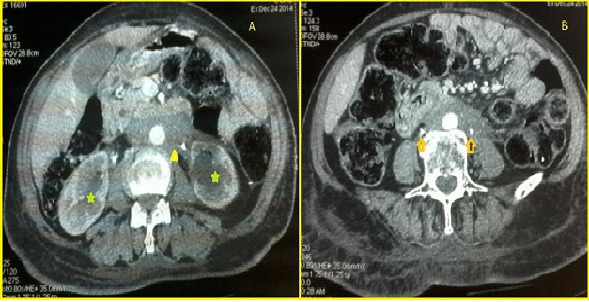
A) (tête de flèche jaune) uro scanner: présence d'un manchon fibreux péri-aortique de densité tissulaire, homogène, s’étendant de façon asymétrique autour des deux uretères lombaires et entraînant; B) (flèche) une urétéro-hydronéphrose plus marquée à gauche (étoiles vertes)

